# Verifying calculated dose to the breast in stereotactic radiotherapy: A practical phantom‐based method

**DOI:** 10.1002/acm2.70841

**Published:** 2026-10-09

**Authors:** Stewart J. Becker, Ryan E. Smith, Wesley S. Culberson, Kurt E. Stump, Baoshe Zhang, Jie Ding, Sina Mossahebi, Dennis Herschbach, Elizabeth M. Nichols

**Affiliations:** ^1^ Department of Radiation Oncology University of Maryland School of Medicine Baltimore Maryland USA; ^2^ Office of Research and Development University of Maryland, Baltimore Baltimore Maryland USA; ^3^ Department of Medical Physics University of Wisconsin ‐ Madison Madison Wisconsin USA; ^4^ Department of Radiation Oncology Mission Cancer Center Asheville North Carolina USA; ^5^ Department of Radiation Oncology Emory University School of Medicine Atlanta Georgia USA

**Keywords:** 3D Printing, breast, GammaPod, quality assurance, SRS, validation

## Abstract

**Background:**

Reference dosimetry for the GammaPod breast radiosurgery device is determined in a polymethyl methacrylate (PMMA) phantom, while the clinical dose calculations assume the patient's breast has the atomic composition of water with a density (∼0.935 g/cm^3^). Direct measurement within this clinical breast‐tissue equivalent material is desirable to validate the treatment planning system (TPS) with end‐to‐end testing and patient‐specific quality assurance (PSQA).

**Purpose:**

This study aimed to validate the GammaPod TPS dose calculations by 3D printing a breast‐equivalent phantom and to develop a practical and reproducible method for end‐to‐end testing and PSQA.

**Methods:**

Multiple breast‐shaped phantoms matching the L02 GammaPod cup geometry were fabricated; three using 3D printing and one using clear ice. The 3D‐printed phantoms were constructed from polylactic acid (PLA) and premium PLA (PLApro) using optimized infill densities to achieve a physical density near 0.935 g/cm^3^, matching the TPS's breast‐tissue model. These infills were determined by creating series of CT phantom plugs and choosing the ones with the closest Hounsfield units (HUs) and stopping power ratios (SPRs) to the TPS breast tissue model. A rotating insert design allowed multi‐position measurements with an Exradin A1SL ionization chamber and thermoluminescent dosimeters (TLDs). CT imaging and dosimetry were performed following the clinical GammaPod workflow. An ice phantom, with a density of approximately 0.918 g/cm^3^, served as an independent validation reference. Measured doses were compared with TPS‐calculated doses.

**Results:**

The breast‐tissue mimicking phantoms yielded mean Hounsfield (HU) and Stopping Power Ratios (SPR) numbers of −81 HU ± 5 and 0.99 SPR for blue PLApro (92.5%), −69 HU ± 11 and 0.99 SPR for natural PLA (86.0%), −82 HU ± 5 and 1.00 SPR for the ice phantom corresponding to densities of 0.916 g/cm^3^, 0.928 g/cm^3^, and 0.915 g/cm^3^ respectively, comparable to the modeled breast tissue. Measured‐to‐calculated dose differences for the ice phantom were within ± 0.5%, while the 3D printed phantoms had an average difference of 1.2%, consistent with published dosimetric uncertainty limits of the GammaPod. The total estimated measurement uncertainty, including charge‐to‐dose conversion and material variability, was 1.7%. The 3D‐printed phantoms demonstrated excellent reproducibility when printing parameters were held constant.

**Conclusion:**

The GammaPod TPS accurately predicts dose in breast‐equivalent materials. The 3D‐printed breast phantom provides a reliable, water‐composition breast‐density material, and geometrically accurate tool for end‐to‐end testing and PSQA, eliminating the challenges associated with water measurements. This method supports routine clinical verification and offers a reproducible framework for ongoing GammaPod QA and commissioning.

## 1 INTRODUCTION

The GammaPod (Xcision Medical Systems; Columbia, MD) is a novel, breast‐specific stereotactic radiosurgery device designed for prone‐position treatment of breast cancer. It employs twenty‐five ^60^Co sources arranged in a bowl‐shaped configuration that rotate around a common isocenter, delivering radiation through 15‐ and 25‐mm collimators.

Patient immobilization and stereotactic localization are achieved with a specialized cup system consisting of an inner cup, an outer cup, and a silicone flange. The inner cup contacts the breast directly; the outer cup secures the patient to the treatment table; and the flange creates an airtight seal between the cups and the skin. Similar in concept to the Gamma Knife frame, the GammaPod cup system uses a vacuum rather than fixation pins. The vacuum, applied between the inner and outer cups, gently pulls the breast away from the chest wall through small holes in the inner cup, holding it in a stable, reproducible position. The outer cup contains a stereotactic wire for localization and locks the assembly to the treatment table.

This cup system not only provides immobilization and localization but also constrains the breast to a finite set of geometric configurations. Cups are defined by two diameters (medium or large) and ten depths each, resulting in twenty available geometries. This limited number of fixed shapes enables the use of pre‐calculated Monte Carlo dose kernels, greatly reducing time needed for planning by removing the calculation phase and only requiring optimization on the target geometry. Previous studies have demonstrated the accuracy of this cup system, its reduced planning time, workflow, and its clinical utility.^1^, ^2^, ^3^, ^4^, ^5^, ^6^, ^7^, ^8^

As described by Becker et al.^9^ and Yi et al.,^10^ reference dosimetry for the GammaPod is performed in a polymethyl methacrylate (PMMA) phantom slightly larger than the largest cup. The dose rate measured in this PMMA phantom serves as the reference for the treatment planning system (TPS). For clinical planning, the TPS assumes that all tissue within the cup has the elemental composition of water but a density of 0.935 g/cm^3^, corresponding to typical breast tissue. The nominal dose rate of the GammaPod ranges from 1.5‐3.5 Gy/min depending on the ages of the sources. Monte Carlo simulations are used to convert reference dosimetry from PMMA to this “breast‐equivalent” material. Becker et al.^5^ further validated this approach experimentally, although their method was time‐consuming and required a correction for density differences between water and breast tissue.

The goal of this study was to validate the dose calculated to the patient by the GammaPod TPS and to establish a practical method for end‐to‐end testing and patient‐specific quality assurance (PSQA). To further this goal, we designed and fabricated three phantoms: one ice phantom to independently validate the calculated dose and other two 3D‐printed breast phantoms (different material) to create a more practical version. Both were matched to the geometry of a commonly used GammaPod cup (L02, large diameter with the third‐smallest depth) and were designed to fit directly into the corresponding outer cup without the flange or inner cup. A fourth cup was also created with a purposely lower density to determine the sensitivity of dose to HU. An early prototype was presented in an AAPM conference abstract.^11^ This manuscript expands upon the design while testing new material and includes more comprehensive methods of validating the results.

## 2 MATERIALS AND METHODS

### 2.1 Phantom requirements

A GammaPod phantom must satisfy three key requirements to evaluate the TPS‐calculated dose:

1. Geometric compatibility: It must match the size and shape of one of the inner cups, replicating a patient's breast. Separate phantoms are required for each cup size. Figure 1 shows the cup system components and a CT image of a patient fitted with an L02 cup (large diameter, third‐smallest depth).
2. Measurement capability: It must accommodate ionization chambers and thermoluminescent dosimeters (TLDs at multiple locations).
3. Material equivalence: The phantom must have an appropriate physical density and atomic composition. The TPS models breast tissue as water‐equivalent (Z_eff_ ≈ 7.42) with a density of 0.935 g/cm^3^.

**FIGURE 1:**
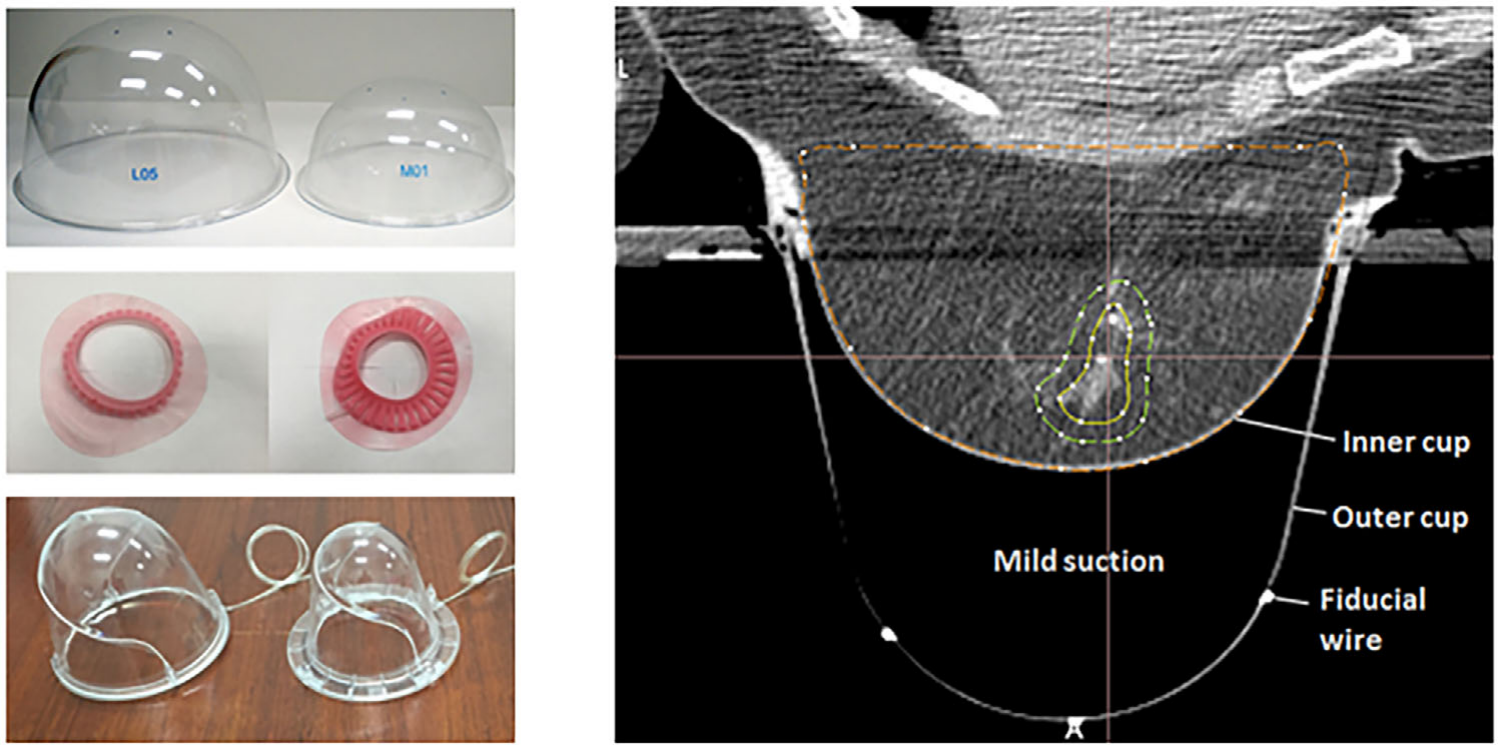
(Left) GammaPod cup system: inner cup (top), flange (middle), and outer cup (bottom). (Right) CT image of breast positioned in L02 cup.

### 2.2 Phantom design

The breast phantom was designed to meet these requirements: matching the L02 cup geometry, mimicking the TPS material assumptions, and allowing multi‐point and multi‐dosimeter dose measurements. The L02 cup geometry was imported into Fusion computer‐aided design (CAD) software (Autodesk, San Franscico, USA), and the phantom was modeled to fit securely within the large outer cup. This is an improvement of the previous design from Becker et al which required the use of the inner cup and flange. The new design greatly simplifies the setup and makes it sit more securely in the machine producing more accurate measurements.

To enable multi‐position dose measurements without an excessive number of holes, we implemented a rotating insert design. A single large insert with a ion chamber hole was offset from the phantom's center. By rotating the insert, measurements could be taken at predefined radii and angles. Chamber depth was varied using interchangeable holders that positioned an Exradin A1SL ionization chamber (Standard Imaging, Middleton, WI) at different depths. Additional inserts were designed to hold IROC‐Houston TLDs. Figure 2 illustrates the phantom, rotating insert, and chamber holder. The insert design was improved over the previous results in the conference abstract which resulted in a smaller air gap between itself and the main body of the phantom. The ease of rotation was also improved as a result.

**FIGURE 2:**
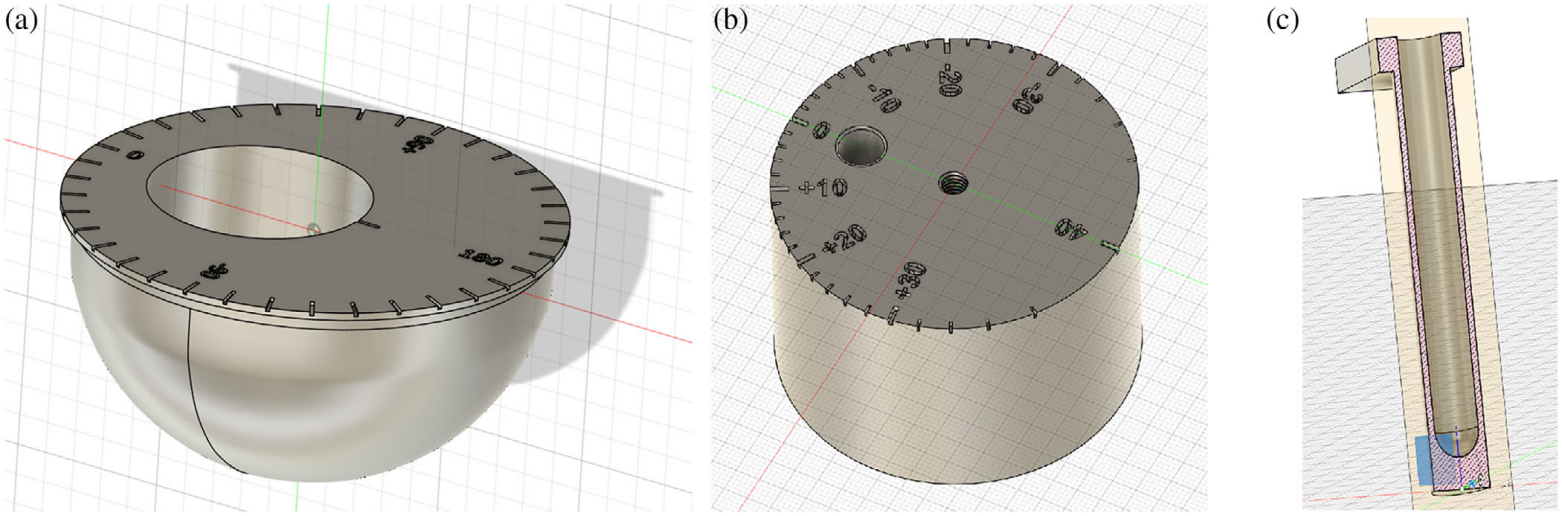
CAD models of (a) phantom body, (b) rotating insert, and (c) cross‐section of chamber holder.

Material selection and 3D‐printing parameters such as infill pattern and percentage were based on prior studies by Dancewicz et al.,^12^ Kumar et al.,^13^ and Fonseca et al.^14^ We evaluated three common filaments—polylactic acid (PLA) (HATCHBOX 3D, Los Angeles, CA), reinforced PLA (PLApro) (3D‐Fuel, Fargo, ND) and acrylonitrile butadiene styrene (ABS) (HATCHBOX 3D, Los Angeles, CA) —printed with a Prusa MK4S printer (Prusa Research; Prague, Czech Republic). Cylindrical test samples were printed with various infill percentages and patterns, then scanned in a CT electron density phantom (Sun Nuclear, FL). Based off published literature, a gyroid infill pattern was chosen for its uniform density and isotropic response to multiple beam angles.^15^ This was an additional improvement over the rectilinear pattern used in previous work.

The cylinders were scanned using single‐energy (Somatom go.Open.Pro) and dual‐energy (Somatom Definition Edge) CT scanners (Siemens Healthineers, Erlangen, Germany). The following parameters were used for the single and dual energy CT scans, respectively: tube voltage, 120kVp, 80 kVp, and 140 kVp; effective tube current‐time product, 300 effective mAs, 400 effective mAs, and 95 effective mAs; pitch, 1.2 for all energies; slice thickness, 1 mm for all energies. The resulting Hounsfield units (HUs) and effective atomic number (Z_eff_) were used to determine the optimal infill and material combination. DirectSPR software/application of Siemens Healthineers is designed for dual‐energy CT (DECT) to directly estimate the stopping power ratio (SPR) of tissues without using conventional HU‐to‐SPR conversion curves which are used in single‐energy CT.

When we acquire CT data at two different X‐ray energy spectra (80 and 140 kVp), DirectSPR uses material decomposition algorithms to determine the tissue's electron density (*ρ*_e_) and Z_eff_. These quantities are then used in a physics‐based model derived from the Bethe‐Bloch equation to calculate the SPR for each voxel directly.

Based on these results, three full‐scale GammaPod L02 phantoms were printed: two from blue PLApro (86.0% and 92.5% gyroid infill) and one from natural PLA (86.0% gyroid infill). The 92.5 % infill blue PLApro phantom was based off the CT plug data. The 86.0% infill blue PLApro phantom was created to test how the sensitivity of dose to HU. Each phantom underwent the standard GammaPod imaging workflow, as described previously.^2^ The CT images were imported into the TPS, verified for dimensional accuracy, and used to create treatment plans targeting multiple internal points. Since this phantom is water equivalent unlike the standard PMMA GammaPod phantom, measured charge can be converted to dose by using IAEA–AAPM TRS‐483^16^ and N_D,w_ in the formula below.

$$D_{w,Q_{msr}}^{f_{msr}}=M_{Q_{msr}}^{f_{msr}}*N_{D,w,Q_{0}}^{f_{ref}}*k_{Q_{msr}Q_{0}}^{f_{msr}f_{ref}},$$

where

$D_{w,\;Q_{msr}}^{f_{msr}}$: The absorbed dose to water at the reference depth in the machine‐specific reference field

$M_{Q_{msr}}^{f_{msr}}:$ The fully corrected charge from the ion chamber under the measurement conditions.

$N_{D,w,Q_{0}}:$ The calibration factor in terms of absorbed dose to water for the ion chamber, obtained from a standards laboratory using a standard reference quality Q_0_. In this case Co60

$k_{Q_{msr},Q_{0}}^{f_{msr},f_{ref}}:$ The correction factor that accounts for the differences between the machine‐specific reference field ($f_{msr})$ and the standard calibration conditions ($f_{ref}$) and beam quality *Q*_0_. Based off results from TRS 483 and Yi et al, $k_{Q_{msr},Q_{0}}^{f_{msr},f_{ref}}$ can be assumed to be unity for the 25 mm collimator.

Using this formula we convert the collected charges to dose which were then compared directly to the TPS calculated values.

### 2.3 Validation

Validation of the TPS was performed using two separate methods. First, a TLD supplied by the IROC dosimetry services was placed in a specially designed holder in the blue PLApro phantom and irradiated with a single isocenter. Second, a shell replica was created and filled with water, which was then frozen to form an ice phantom. Ice provides an excellent physical surrogate for the breast material modeled in the TPS, with identical elemental composition (H_2_O) and a density of approximately 0.918 g/cm^3^—closely matching the TPS's 0.935 g/cm^3^ assumption.

Clear, bubble‐free ice was achieved via directional freezing, in which water freezes from one controlled direction, forcing air bubbles out and producing optically clear ice. A 6.35‐mm (¼‐inch) hole—matching the A1SL chamber diameter—was drilled to the same depth used in the printed phantom experiments. The ice phantom was imaged and irradiated using identical treatment plans as the printed phantoms. Measured doses were compared directly to TPS calculations.

## 3 RESULTS

Fourteen cylindrical test inserts of various materials, colors, and infill % were fabricated and imaged, yielding a range of HU and SPR values (Table 1). These values helped guide the creation full phantom by indicating which materials had the best SPR and what infill % was needed to create an HU closest to 0.935 g/cm^3^. HU–infill relationships indicated that densities approximating 0.935 g/cm^3^ were achieved at 92.5% infill for blue PLApro and 86% for natural PLA, with average SPRs to water of 0.99 for both.

**TABLE 1:** CT data from multiple inserts materials and infill %.

Material	Color	Infill %	Min HU	Avg HU	Max HU	Interpolated Infill % for Breast Density	SPR to Water
PLA	True Pink	86	−139	−76.7	−1	87.0	0.90
PLA	True Pink	88	−127	−53.7	19		0.91
PLA	True Pink	90	−94	−31.4	31		0.93
ABS	True Pink	86	−301	−237.9	−172	109.5	0.79
ABS	True Pink	94	−245	−179.0	−105		0.85
PLA	Blue	88	−159	−83.4	−18	89.9	0.98
PLA	Blue	90	−120	−63.1	7		0.99
PLA	Blue	92	−108	−45.3	8		1.00
PLA	Black	84	−142	−77.9	8	85.5	0.88
PLA	Black	86	−151	−60.6	29		0.90
Pro PLA	Blue	92	−141	−69.7	26	92.5	0.98
Pro PLA	Blue	94	−126	−49.8	34		0.99
PLA	Natural	84	−157	−82.6	−13	86.1	0.99
PLA	Natural	86	−147	−65.5	16		0.99

Two materials were chosen to create full‐sized L02 phantoms, blue PLApro and natural PLA.

The resulting printed phantoms along with the ice phantom achieved HU close to their target values, mean CT numbers of −81 ± 5 (blue PLApro), −69 ± 11 (natural PLA), and −82 ± 5 (ice phantom), corresponding to physical densities of 0.916, 0.928, and 0.915 g/cm^3^, respectively. The 86.0% blue PLApro phantom had a mean CT numbers of −146 ± 7 corresponding to physical density of 0.848 g/cm^3^.

Figure 3 shows the CT electron density scan and the ice‐filled phantom. Figure 4 shows representative axial and coronal CT images of the blue PLApro phantom.

**FIGURE 3:**
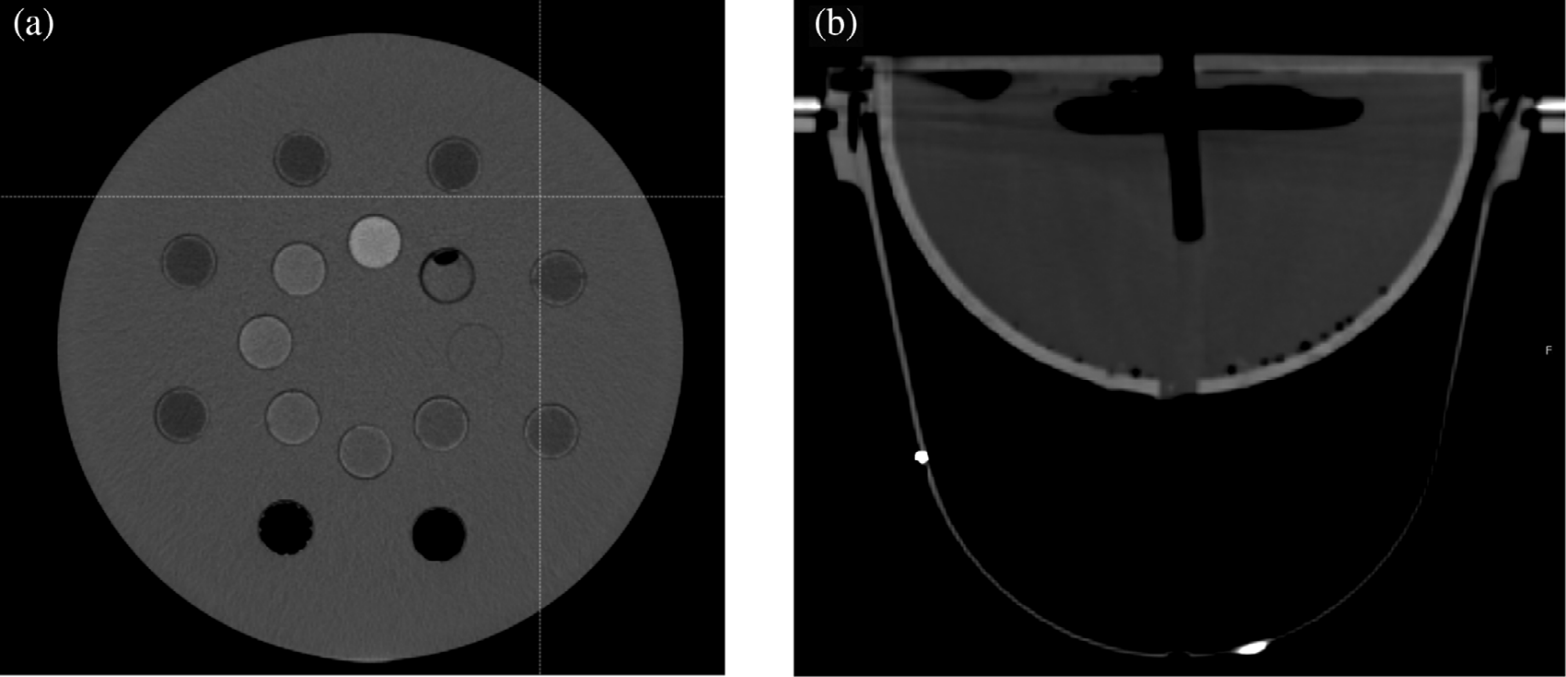
CT images of the a) electron density phantom and b) ice‐filled L02 GammaPod phantom.

**FIGURE 4:**
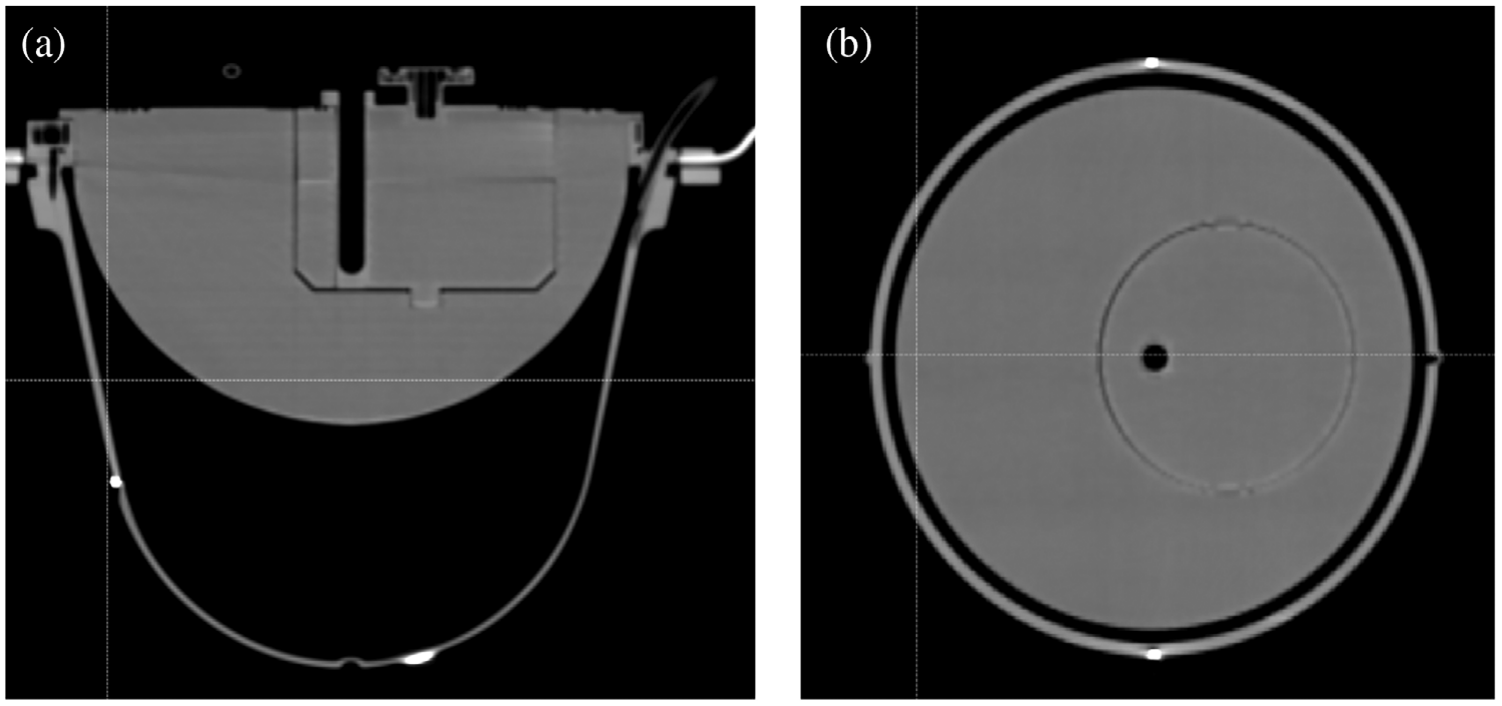
CT images of the breast‐equivalent phantom (Blue PLApro 92.5% infill) a) axial b) coronal.

Multiple treatment plans were delivered to multiple measurement points in both printed phantoms, and to the central point in the ice phantom. Four plans were created and measured in multiple locations. These plans varied in size and location throughout the breast. From larger volumes covering many quadrants typical of boost or APBI volumes to small targets used in single fraction SRS. These attributes are shown in Table 2. The TPS reports a dose averaged over a ∼0.076cc vol (∼5 mm diameter) along with (x,y,z) coordinates. Measured doses agreed closely with TPS calculations (Table 3). The results from the TLD for the single isocenter plan agreed within 1% of TPS dose. The plans 2 & 3 were also run on the 86.0% infill blue PLApro phantom. Compared to the 92.5% blue PLApro phantom, the dose on average was higher by 0.8%. Based off the HU difference between the two PLApro phantoms, this difference translates into a 1% dose difference per 81 HU. This is similar to previously reported Gamma Knife studies which looked into the effect of dose rate based off headsize.^17^

**TABLE 2:** Plan properties. All plans were prescribed so that 95% of target received 3 Gy.

Plan #	Target Vol (cc)	# Control Points	Time (min)	Conformality Index (CI)	Location of target in breast
1	29.0	436	8.63	1.1	Central and lateral
2	35.6	305	6.79	1.1	Central to nipple and lateral
3	0.3	54	2.10	8.7	Central
4	13.7	275	4.99	1.1	Central from nipple to chestwall

**TABLE 3:** Comparison of calculated and measured doses in multiple phantoms. All measurement points were at the same depth but at varying lateral distances. Point A was at the center, with each subsequent point displaced 10 mm further laterally. Only one point in ice phantom was measurable.

			% Difference TPS—Measured		
Plan #	Lateral Position (mm)	Vertical Position (mm)	ProPLA—Blue	PLA—Natural	Ice
1	0	30	−1.4%	−0.4%	0.0%
1	10	30	−1.3%	0.5%	
1	20	30	−1.0%	−0.9%	
1	30	30	0.7%	−0.6%	
1	40	30	−0.6%	−0.3%	
2	0	30	−1.6%	−1.8%	−0.1%
2	10	30	−1.9%	−2.0%	
2	20	30	−2.5%	−2.8%	
2	30	30	−1.1%	−1.8%	
2	40	30	−1.6%	−2.1%	
3	0	30	−1.0%	−0.8%	0.5%
4	0	30	−0.6%	0.2%	
4	10	30	−0.8%	0.4%	
4	0	35	−1.0%	0.2%	
4	10	35	−0.9%	1.3%	
Mean			−1.2%	−1.2%	−0.1%
SD			0.8%	0.9%	0.26%

## 4 DISCUSSION

This work lays out practical method for verifying the dose to the breast for a breast stereotactic radiosurgery by using a 3D printed phantom to mimic breast tissue. The work is much more thorough investigation of a previously published abstract. This work performs a detailed investigation of multiple materials and their associated HUs and Z_eff_. Then by using this data we created multiple breast phantoms which are easier to use and more accurate than previous models. In addition, TLDs have been used to validate the dose along with a novel ice phantom to mimic the breast‐equivalent tissue used by the TPS. These two methods confirmed that the GammaPod TPS accurately converts PMMA‐based calibration to dose in breast‐equivalent material. Measurements from multiple plans in various location throughout the phantom agreed with the TPS, consistent with previously reported uncertainties.^10^

There are various sources of uncertainty in this work. First is the previous published 1.6% dosimetric uncertainty from the charge‐to‐dose conversion (Yi et al.^10^). We followed this same methodology in our study with the same equipment. Additional uncertainty arises from the production of 3D printed phantoms. This includes consistency of prints where we saw a range of ± 16 HU and the effect on HU by color and material which can cause a variation of ± 20 HU according to Hofmann et al.^18^ Our studies show that for PLApro a change in HU of 81 results in a dose difference of 1.0%. The total print variation of ± 36 HU therefore produces a dose uncertainty of 0.4%. Combined in quadrature, the total uncertainty was 1.7%. The difference of 15 HU between the target HU for the blue PLApro phantom and its actual HU affects the resulting dose by just 0.2%. This linear relationship between dose and HU has only been show to be true for this limited infill range and is not assumed to be valid through the entire infill range of 0%–100%.

The 3D‐printed breast phantoms demonstrated that additive manufacturing can reproducibly create tissue‐equivalent phantoms suitable for GammaPod dosimetry, provided that printer type, filament, and print conditions remain consistent. Although the blue and natural PLA filaments required slightly different infill percentages, both achieved similar HU values, Z_eff_, SPRs, and measured‐to‐calculated dose ratios. This is acceptable for end‐to‐end and constancy testing applications, though might not be for reference dosimetry.

While color, manufacturer, and printer settings can significantly affect HU and attenuation properties. If one is consistent in printing and ensuring conditions are well controlled, inter‐print variation is minimal. Our results confirm that consistent 3D‐printing parameters yield reproducible dosimetric performance suitable for QA. Small air pockets were observed at the top of the ice phantom; however, since the GammaPod delivers radiation from below, their dosimetric effect was negligible. Chamber misalignment from drilling was accounted for during dose comparison.

This work expands substantially on our previous efforts in several key respects. First, we broadened our investigation of filament materials to include PLA and PLApro, both of which more closely approximate the atomic composition and effective atomic number (Z_eff_) of water than materials used previously. Second, the phantom design was revised: the phantom now seats directly on the outer cup, eliminating reliance on the inner cup and flange and enabling easier, more precise placement. The rotating insert and chamber holder were also redesigned for improved printability, better component fit, and support for multiple depth positions. Third, prints were produced on a newer printer offering greater reliability and dimensional accuracy compared to our earlier setup. Fourth, slicing parameters were refined based on current literature and newly collected data, with adjustments to infill percentage and infill pattern to more closely mimic the target material and produce more homogeneous prints. Fifth, we performed independent validation through IROC services, as well as through a separate, novel ice phantom selected specifically because it closely replicates both the Z_eff_ and the target density (0.935 g/cm^3^) of water. Sixth, we further used dual‐energy CT to directly assess the Z_eff_ of the printed material, providing an additional, independent means of confirming water equivalence. Finally, this study substantially increased the number of treated plans and measurement points evaluated, encompassing a broader range of plan types, tumor locations, and tumor sizes than previously reported.

## 5 CONCLUSION

We developed and validated the first phantom capable of verifying clinical dose calculations for the GammaPod system. The 3D‐printed breast phantom accurately reproduces the geometry and dosimetric properties assumed in the TPS and provides a safe, practical alternative to water phantoms for end‐to‐end and PSQA testing.

Water phantoms are suboptimal for GammaPod QA due to their different density (1.0 g/cm^3^ vs. 0.935 g/cm^3^), handling difficulties, and spill risk around the open‐source housing. The printed breast‐equivalent plastic phantom mitigates these issues while maintaining dosimetric accuracy. Although other materials may better approximate water in the future, this approach offers a robust and reproducible method for GammaPod QA.

## AUTHOR CONTRIBUTIONS

All authors contributed to either drafting or critical editing of the manuscript's intellectual content well as final approval of the documents. Stewart Becker, Wesley Culberson, Kurt Stump, and Elizabeth Nichols made substantial contributions to design of the work. Stewart Becker, Jie Ding, Ryan Smith, Baoshe Zhang, Dennis Herschbach, and Sina Mossahebi made substantial contribution to data acquisition, printing design, and analysis. Stewart Becker, Wesley Culberson, Kurt E. Stump, and Elizabeth Nichols made substantial contributions to interpretation of data for the work.

## CONFLICT OF INTEREST STATEMENT

Stewart J. Becker, PhD, is the owner of Revolution Physics LLC, a company in the process of developing and manufacturing the BEST phantom (Breast‐Equivalent Simulation Tool), the device described in this work. This relationship represents a potential conflict of interest and has been disclosed in accordance with journal policy.

## ETHIC STATEMENT

This article does not contain any studies with human participants or animals performed by any of the authors. Institutional Review Board (IRB) approval was not required for this non‐clinical study.

## Data Availability

The datasets generated and/or analyzed during the current study are available from the corresponding author on reasonable request.
